# Fucose as a new therapeutic target in renal transplantation

**DOI:** 10.1007/s00467-020-04588-2

**Published:** 2020-05-29

**Authors:** Mark C Howard, Christopher L Nauser, Daniela A Vizitiu, Steven H Sacks

**Affiliations:** 1 MRC Centre for Transplantation, Peter Gorer Department of Immunobiology, School of Immunology & Microbial Sciences, King’s College London, London, UK; 2 King’s College London, London, UK

**Keywords:** Ischaemia/reperfusion, Complement, Lectin pathway, Collectin-11, Fucose

## Abstract

Ischaemia/reperfusion injury (IRI) is an inevitable and damaging consequence of the process of kidney transplantation, ultimately leading to delayed graft function and increased risk of graft loss. A key driver of this adverse reaction in kidneys is activation of the complement system, an important part of the innate immune system. This activation causes deposition of complement C3 on renal tubules as well as infiltration of immune cells and ultimately damage to the tubules resulting in reduced kidney function. Collectin-11 (CL-11) is a pattern recognition molecule of the lectin pathway of complement. CL-11 binds to a ligand that is exposed on the renal tubules by the stress caused by IRI, and through attached proteases, CL-11 activates complement and this contributes to the consequences outlined above. Recent work in our lab has shown that this damage-associated ligand contains a fucose residue that aids CL-11 binding and promotes complement activation. In this review, we will discuss the clinical context of renal transplantation, the relevance of the complement system in IRI, and outline the evidence for the role of CL-11 binding to a fucosylated ligand in IRI as well as its downstream effects. Finally, we will detail the simple but elegant theory that increasing the level of free fucose in the kidney acts as a decoy molecule, greatly reducing the clinical consequences of IRI mediated by CL-11.

Ischaemia/reperfusion injury (IRI) is a well-known consequence of transplantation. This process has been studied extensively in various systems, but the focus of our research has been on the kidney and the role of complement in the injury mechanism. Recent work has shown that signalling by collectin-11 (CL-11) is key to the activation of the complement system and is a major contributor to the renal injury caused by ischaemia-reperfusion insult. Furthermore, preliminary experiments have suggested that a fucosylated ligand, exposed in the process of IRI, is a trigger for this CL-11-mediated activation of the complement cascade [1]. In this review, we will briefly summarise the significance of IRI in renal transplantation, describe the complement system and the biological relevance of fucose as a trigger factor and then consider future treatment strategies arising from these observations on fucose and CL-11.

## Renal transplantation

At the time of writing of this manuscript, 4910 people in the UK and 94,878 people in the US are awaiting kidney transplantation. It has been shown that successful renal transplantation reduces the mortality of patients receiving a transplant versus those remaining on dialysis [2]. Furthermore, there is a measurable improvement in the quality of life for kidney transplant recipients compared with dialysis patients [3]. Despite the lifesaving and lifegiving potential that organ transplantation allows, there are some unavoidable consequences, ranging from immediate technical and surgical issues to the need for long-term immunosuppression. One issue that is particularly relevant to this review is IRI, which is increasingly important in organs procured from deceased donors (Deceased Donor Renal Transplants; DDRT). Deceased donor organ transplantation is the mainstay of transplants carried out and accounts for more than 50% of transplants in the US [4]. However, it has been well established that living donor kidney transplants have lower graft failure rates at 6 months and 10 years as well as commensurate improvements in patient survival [5]. Furthermore, living donor renal transplants last about 25% longer and are associated with better overall survival than DDRTs [6]. These findings may be due in part to the early actions and response of the immune system. Interestingly, allograft survival from living donors is less influenced by HLA matching than due to decreased ischaemic injury, which is known to influence and activate the alloimmune response [7]. Underpinning the body’s response to transplantation is the immune system, which begins with the immediate innate immune response. In particular, the complement system begins exerting its action immediately and has long-term implications of its effects, which we will discuss throughout this review. For example, we now know that the complement system augments T cell and B cell mediated alloimmunity [8, 9] and is necessary to prime antibody production against donor tissue [10]. What is more, complement likely exerts its role in the vascular compartment as well as the extravascular compartment of the donor organ. Indeed, clinical trials are underway looking at the use of C1 INH and eculizumab in the treatment and prevention of antibody mediated rejection (ABMR) in solid organ transplants [11, 12]. Thus, unravelling and mitigating the early immune response to organ transplantation could have a profound impact on overall graft and patient survival.

## The complement system

As stated above, the complement system has been implicated in the detrimental effect of IRI on renal transplantation. The complement system is a crucial part of the innate immune system which, through pattern recognition, not only acts in host defence and to remove immune complexes and cell debris, but it also acts to coordinate inflammation and recruit the adaptive immune system [13]. Complement activation occurs through three main routes: the classical, lectin and alternative pathways. The classical and lectin pathways require triggering by pattern recognition molecules (PRMs), while the alternative pathway is activated by cleavage of C3. All three pathways converge to cleave C3, which causes important downstream effects of complement including formation of the membrane attack complex (MAC) [14]. Whereas the PRM of the classical pathway, C1q, triggers complement activation in ABMR against donor specific HLA antigens [15], renal IRI is classical pathway-independent [16, 17]. Therefore, the rest of this review will concentrate on the lectin and alternative pathways.

The lectin pathway has a large number of PRMs including ficolins and collectins [reviewed in [ [18]]. These are C-type lectins (CTLs), which mean their binding to carbohydrate ligands is calcium-dependent. Briefly, ficolins contain a fibrinogen-like domain and a collagen-like domain, acting as opsonins as well as forming complexes to activate the complement pathway [19]. Collectins, also CTLs, are a family of nine molecules containing a collagen-like domain and a carbohydrate recognition domain (CRD) responsible for pattern recognition [18]. The most relevant collectins to renal IRI are mannose binding lectin (MBL) and collectin-11 (CL-11). MBL is expressed in the liver in humans and is present in serum as a trimer [20]. MBL binds with high affinity to D-mannose and GlcNAc and lower affinity with ligands such as galactose [21, 22]. In contrast, CL-11 binds with a higher affinity to ligands such as L-fucose and D-mannose than with those such as D-galactose and D-glucose [23]. For example, CL-11 binds to a subgroup of fucosylated ligands such as Lewis^a^ and Lewis^y^ in a process that is again Ca2+ dependent [24]. CL-11 is expressed in numerous cell types throughout the body and alongside collectin-10 (CL-10) as a heterodimer. This has predominantly been shown in the circulation, but CL-10 mRNA and protein have been detected in the kidney, amongst many other organs [20, 25]. Thus, it could be considered that MBL is a guardian of the intravascular compartment, whereas CL-11 is sited in the extravascular compartment where it is produced locally by many tissues.

CL-11, like MBL, is physically associated with serine proteases known as MBL-associated serine proteases (MASPs 1–3). Once CL-11 has bound to its target, the CL-11/MASP complex initiates complement activation. Traditionally, this was thought to be through cleavage of C4 and C2 to produce the C3 convertase C4b2a [14]; however, more recent work has shown the presence of a C4 bypass pathway where the CL-11/MASP complex can directly cleave C3 [26]. Cleavage of C3 causes the release of the main effectors of the complement system: notably C5a and C3a (which mediate anaphylaxis), C3b and C3d (which mediate cell-cell adhesion and opsonisation) and MAC [13]. MAC (membrane attack complex) is composed of C5b, C6, C7, C8 and C9 (C5b-9) and is formed on the target cell surface upon activation of the terminal complement pathway. These components create a transmembrane pore that forces the cell into osmotic lysis [27]. Nucleated cells are relatively well protected against cell lysis by MAC, and cell activation associated with calcium influx and mobilisation due to MAC formation is associated with a proinflammatory state. It is important to note that since PRMs of the complement system are produced both locally and systemically, in any given location of complement activation, there could be a complex interplay between locally made components and those that diffuse into the tissue from the serum. However, it is conceivable that tissue production has a predominant effect at the site of localised tissue stress or infection, whereas the circulating pool provides an important backstop in the case of invasive pathogens [18].

**Fig. 1:**
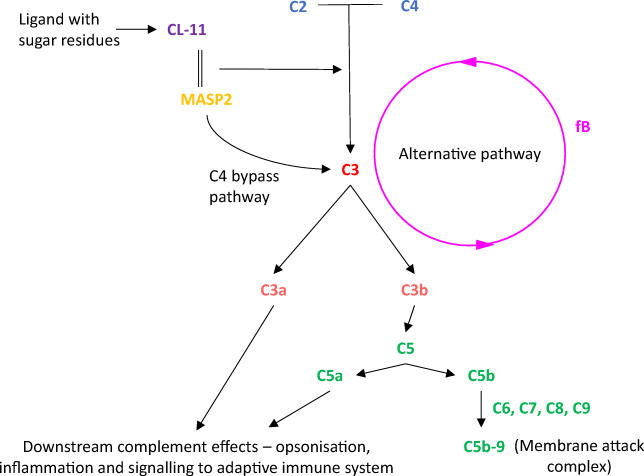
Key relevant components of the complement system. A simplified diagram of the complement system showing the pathways and factors that are relevant to this review. CL-11 binds to a sugar residue on a target molecule, such as those on bacteria or exposed on damaged tissue, and alongside the serine protease MASP2 cleaves C3. This is either through two other complement components C2 and C4 which once cleaved form a C3 convertase, or it is directly through the C4 bypass pathway. The alternative pathway uses the cleavage fragment C3b to make another C3 convertase, a process in which fB is essential. There is a low level of spontaneous cleavage of C3, termed tickover, at all times but upon activation of C3 by the classical pathway or lectin pathway the rate of cleavage of further cleavage of C3 via the alternative pathway increases rapidly. This amplifies the ultimate downstream effects of the complement system, be they opsonisation by C3b, inflammation by C3a and C5a, or membrane injury by the formation of the membrane attack complex (C5b-9). Furthermore, these downstream components, specifically C3a, C5a and C3b, signal to, and recruit the adaptive immune system

By contrast with the lectin and classical pathways of complement activation, spontaneous hydrolysis of C3 is thought to provide the alternative pathway with a continuous low level of activity leading to C3b formation (tickover). The alternative pathway can also function as an exponential feedback loop where the cleavage product C3b, formed by activation of the classical or lectin pathways, recruits the alternative pathway proteins factor D (fD) and factor B (fB). fD cleaves fB into Bb. Following this, C3bBb formed from C3b and Bb is the alternative C3 convertase, cleaving C3, creating more C3b (and C3a) and so on. Importantly, the cleaved thioester bond of C3 reacts with cell surfaces keeping this alternative pathway activation local to the activating cell surface [28]. The components of the complement system that are pertinent to this review are detailed in Fig. 1.

## The complement system in IRI

Although early reperfusion is essential for salvaging cells with reversible ischaemic damage, reperfusion following prolonged ischaemia may induce injury to ischaemic tissue, resulting in cellular damage and cell death [29], mainly involving the renal tubular epithelial cells in the case of kidney transplants. Rapid activation of complement upon reperfusion of the ischaemic kidney promotes C5a-mediated neutrophil infiltration and oxidative stress (via reperfusion-induced oxygen-free radical formation) as well as direct membrane injury through MAC formation [16].

Murine renal IRI models can mimic this property of kidney transplantation after occlusion of the main renal arteries and veins for a variable period of time (ischaemia) followed by a period of reoxygenation of the renal tissue by allowing blood flow back into the tissues (reperfusion). This results in complement-mediated renal damage, further enhanced through activation of downstream signalling pathways involved in innate immunity [16]. By allowing this process to take place, IRI provides a reproducible and quantifiable model useful to study the complement system and how its effects can be diminished during renal transplantation [1, 16, 30, 31]. This is an especially important model because it lends itself to the study of prolonged ischaemic times, where organs are more prone to undergo renal damage with a decreased chance of survival and a delayed graft function [32].

As implied earlier, the lectin and alternative pathways are the main complement pathways activated during renal IRI. There is increasing evidence that PRMs, normally reacting with surface-bound carbohydrates on pathogens, during IRI bind to endogenous ligands expressed on the surface of injured cells, such as those found in post-ischaemic kidney as well as phospholipids and nucleic acids [33].

C3 deposition along the renal tubular basement membrane then occurs following the activation of the complement system. During this activation, levels of circulating C3a may also be increased [33]. While hepatocyte synthesis is the primary source of C3 in the circulation, proximal tubule epithelial cells (PTECs) are involved in local C3 synthesis within transplanted kidney, with a 16% increase in synthesis within an organ undergoing rejection. Regulation of C3 synthesis at this location is mediated by multiple factors, including proinflammatory cytokines [9]. C3 deficiency in mice is protective against renal IRI and also reduces infiltration of immune cells [16], as most of the damage results from an absence of locally made C3 as opposed to circulating C3 [discussed further in [34]]. These data demonstrate that locally produced C3 is essential to complement mediated damage in renal IRI, as well as having a role in the initiation of recruitment of other immune cells [16].

By contrast, experiments in C4-deficient mice have suggested no effect of the classical pathway in renal IRI [16, 17]. This does not, however, exclude an effect of the lectin pathway, since lectin pathway activation to cleave C3 can occur in the absence of C4, explained by lectin-associated serine protease MASP-2 being able to directly cleave C3 [35]. This is further demonstrated in work on transplants in MASP2 and C4 single and combined double knockout mice, where there was no significant increase in protection from renal IRI in mice deficient of both C4/MASP-2 compared to MASP-2^−/−^ alone, in terms of C3d deposition and tubular damage [30]. Furthermore, C1q deficiency in other mouse models of ischaemia failed to show a protective effect [36, 37], suggesting that the classical pathway has no generalised role in the induction of organ reperfusion injury.

Mice with factor B (fB) deficiency (a key component of the alternative pathway) demonstrate very little C3 deposition following IRI, thus demonstrating the importance of the alternative pathway during renal IRI [33]. Similar results emerged from renal ischaemia studies in mice treated with blocking antibody against fB, which showed marked sparing of complement deposition and renal tubule damage in the treated mice and reduction in plasma C3a [31]. Since the alternative pathway can be initiated by spontaneous hydrolysis of C3 or it can serve to amplify the amount of C3b generated by classical or lectin pathways, as mentioned above, and taking into account the evidence in MASP-2^−/−^ mice, it seemed likely that the lectin pathway could be main trigger of complement activation followed by amplification through the alternative pathway.

What remained unclear, however, were the structures that initiated lectin complement activation in ischaemic kidney and how these were recognised. New evidence began to emerge following the discovery of CL-11, in Japan [38]. CL-11 is a lectin pathway PRM that plays a role in antimicrobial defence through its activation of the innate immune system via the lectin complement pathway [23]. Farrar and colleagues (2016) studied the expression of CL-11 within renal tissue after C57BL/6 mice underwent renal vessel ligation followed by reperfusion injury. A rapid increase of CL-11 occurred in the post-ischaemic renal cortex compared to basal levels measured in non-ischaemic kidney, the highest level of CL-11 being found in the renal tubular epithelium within 6 h of reperfusion. CL-11 was localised on the surface of these cells as well as being detected in the cell cytoplasm. Localisation by immunofluorescence of L-fucose, a preferred ligand for CL-11 ligand (whose role is later discussed in this review), was found on the corticomedullary tubule basolateral border in a similar fashion to CL-11 and C3d. Under normal non-ischemic conditions, L-fucose is generally expressed only within cortical tubule segments [1]. Thus, complement activation product (C3d) and a key pattern recognition molecule (CL-11) are found closely aligned on the vulnerable segment of renal tubule, at a site where a key ligand (L-fucose) is selectively expressed. This suggested a trigger mechanism by which tissue stress-associated ligand could lead to binding of CL-11 and lectin pathway activation following renal ischaemia-reperfusion insult.

More definitive support for this hypothesis came from experiments in CL-11-deficient mice. Not only were the mice resistant to the induction of ischaemic injury in native kidney, with reduced complement deposition, tubule cell death and macrophage infiltration, but CL-11-deficient kidney transplanted into wild-type mice with normal systemic production of CL-11 were similarly protected [1]. Locally made CL-11 was therefore largely responsible for mediating the complement attack in this model. For further proof, we needed to specify the role of the renal tubule cell in presenting the fucosylated ligand recognised by CL-11. Let us first consider how and in what form fucosylated ligands are expressed at the mammalian cell surface.

## The role of L-fucose

L-fucose (6-deoxy-L-galactose), a common component of many glycans and glycolipids, is a monosaccharide distinct from other mammalian sugars in its L-configuration [39] and structurally in the lack of a hydroxyl group on the C-6 carbon [40]. The pathophysiological roles for fucosylated molecules are extensive and include a number of diseases including leukocyte deficiency syndrome II (LADII) [41] and tumour progression in certain cancers [42]. Fucosyltransferases, which synthesise fucosylated glycans, are made up of a group of 13 genes, the most studied of which (FUT1 and FUT2) are responsible for H blood group antigen and related structure synthesis [39]. Among these fucosylotransferases, FUT9 also synthesises the Lewis x (le^x^) carbohydrate epitope, which is an important part of the epitope of molecules such as CD15, a differentiation marker of cells [43].

In mammalian oligosaccharides, L-fucose modifications make up 7.2% of the total, indicating wide ranging and important roles in human biology [44]. In this context, fucose can either alter the centre of complex N-glycans, be linked directly to threonine or serine residues or be incorporated into the terminal portions of N-, O- or lipid-linked chains of oligosaccharides [40]. N-glycans are attached to a protein covalently at asparagine (Asn) residues by an N-glycosidic bond, and in all eukaryotic N-glycans contain a GlcNAcβ1-Asn. They then become termed ‘complex N-glycans’ upon the addition of a sugar residue to N-glycan core, the major being α1-6Fuc by α1-6Fucosyltransferase (FUT8). An alternative sugar addition is ‘capping’ where addition of fucose or other molecules such as galactose and GLcNAc are added to complex N-glycan branches. These capping sugars protrude away from the branches due to being α-linked and therefore are presented to potential binding partners such as lectins and antibodies [further detailed in[ [45]]. Fucose can modify two types of O-glycans, mucin O-GalNAc glycans, containing a GalNAc attached to a serine or threonine, or a further subset to which fucose attaches directly to serine or threonine residues [40].

Fucose modifications are controlled by fucoscyltransferases as mentioned above, all of which use GDP-fucose, a nucleotide-activated form of fucose. GDP-fucose acts as a fucose donor in the formation of fucosylated oligosaccharides [39]. Synthesised GDP-fucose is transported into the lumen of the Golgi or endoplasmic reticulum where it can be used by fucosyltransferases [40]. The currently known human fucosyltransferases are listed in Table 1. In these ways, a small alteration in the structure of a molecule such as in this case, adjusting its fucosylation, can alter its location, binding properties and ultimately its function.

**Table 1:** List of currently known fucosyltransferases in humans. The bolded Fucose residue (**Fuc**) represents the fucose moiety added by the listed fucosyltransferase [adapted from[ [ 39, 40]]

FUT (Refseq accession number)	Full name	Representative product (s)	Notes
FUT1 (NM_000148.1)	H blood group α2fucoscyltransferase	**H antigen, type 2** **Fucα2**Galβ4GlcNAc-R	Can add fucose to oligosaccharide chains on N-glycans, mucin O-glycans and glycolipids
FUT2 (NM_000511.1)	Secretor (se) blood group α2fucoscyltransferase	**H antigen, type 1** **Fucα2**Galβ3GlcNAc-R	
FUT3 (NM_000149.1)	Fuc-TIII α3/4fucoscyltransferase Lewis blood group fucosyltransferase	**Sialyl-Lewis**^**x**^ Siaα3Galβ4[**Fucα3**]GlcNAc-R **Sialyl-lewis**^**a**^ Siaα3Galβ3[**Fucα4**]GlcNAc-R **Lewis**^**b**^ Fucα2Galβ3[**Fucα4**]GlcNAc-R **Lewis**^**x**^ Galβ4[**Fucα3**]GlcNAc-R **Lewis**^**a**^ Galβ3[**Fucα4**]GlcNAc-R **Lewis**^**y**^ Fucα2Galβ4[**Fucα3**]GlcNAc-R	
FUT4 (NM_002033.1)	Fuc-TIV α3fucoscyltransferase ELAM-1 ligand fucosyltransferase	Galβ4[**Fucα3**]GlcNAcβ3Galβ4GlcNAc-R Galβ4]GlcNAcβ3Galβ4[**Fucα3**GlcNAc-R Galβ4[**Fucα3**]GlcNAcβ3Galβ4[**Fucα3**]GlcNAc-R SiααGalβ4GlcNAcβ3Galβ4[**Fucα3**]GlcNAc-R Siαα3Galβ4[**Fucα3**]GlcNAc-R	
FUT5 (NM_002034.1)	Fuc-TV α3fucoscyltransferase	Galβ4[**Fucα3**]GlcNAc-R Siaα3Galβ4[**Fucα3**]GlcNAc-R	
FUT6 (NM_000150.1)	Fuc-TVI α3fucoscyltransferase		
FUT7 (NM_004479.1)	Fuc-TVII α3fucoscyltransferase	Siaα3Galβ4[**Fucα3**]GlcNAc-R	
FUT8 (NM_004480.1)	Fuc-TVIII α6fucoscyltransferase	GNGNManβ4GlcNAcβ4[**Fucα6**]GlcNAc-Asn	Only adds fucose to core fucose on N-glycans
FUT9 (NM_006581.1)	Fuc-TIX α3fucoscyltransferase	Galβ4[**Fucα3**]GlcNAc-R	Can add fucose to oligosaccharide chains on N-glycans, mucin O-glycans and glycolipids
FUT10 (NM_032664.2)	Fuc-TX α3fucoscyltransferase	**Unknown**	Putative α3fucoscyltransferases
FUT11 (NM_173540.1)	Fuc-TXI α3fucoscyltransferase	**Unknown**	
FUT12 (POFUT1) (NM_015352.1)	Protein O-fucosyltransferase 1	Siaα3Galβ4GlcNAcβ3**Fucα**–Ser/Thr Siaα6Galβ4GlcNAcβ3**Fucα**–Ser/Thr	Can add an O-fucose to epidermal growth factor (EGF)-like repeats [46]
FUT13 (POFUT2) (NM_015227.1)	Protein O-fucosyltransferase 2	Galβ3**Fucα**–Ser/Thr	Can add O-fucose to thrombospondin type 1 repeats [47]

Against this knowledge, we examined whether removing fucose from the surface of stressed cells could delete the binding ligand for CL-11. A study of renal tubule cells derived by primary culture from normal mouse kidney found that fucosidase treatment of the hypoxic cells effectively prevented CL-11 binding to those cells. Moreover, the treated cells could no longer deposit C3 from serum. This supported the concept that complement activation on hypoxic cells required a hypoxia-induced ligand to bind CL-11 and initiate complement activation by the lectin pathway. The ligand had to be fucosylated to bind CL-11 because control treatment with an irrelevant glycosidase such as galactosidase failed to reduce CL-11 binding and complement activation [1].

A more pragmatic approach perhaps better suited to clinical evaluation was suggested by the ability of soluble sugars to block the carbohydrate recognition site on CL-11 [24]. Farrar et al. found that by pre-treating rCL-11 with soluble L-fucose, this inhibited CL-11 from binding to hypoxic cultured renal tubule cells and led to a marked reduction of complement deposition from serum in the culture medium [1]. In vivo studies then showed that mice receiving a supraphysiological dose of L-fucose intraperitoneally tolerated the treatment well. The treatment was remarkable, since equilibration of free fucose within the renal space to a concentration 10 times the normal level partially protected the mice against loss of renal function induced by IRI. Mice lacking CL-11 gained no additional protection from L-fucose therapy, indicating that the therapeutic effect in this model was largely CL-11 dependent [48].

These in vitro and in vivo findings present a new opportunity for renal transplantation. Coupled with the unmet need through severely ischaemic donor organs being used for transplantation and the recognised effect of complement based on experimental and clinical observations [4–6, 14], these findings with L-fucose suggest a novel and relatively simple approach to block the triggering of inflammation on hypoxia-stressed tissue. Large doses of L-fucose given to patients for other indications have been well tolerated (see below), opening up the possibility of different routes of treatment in the transplant context. Subject to proof-of-concept studies and protocol optimisation in transplanted mice, which are currently in progress, the options are to treat the donor and/or the explanted organ and/or the recipient to maintain blocking concentrations of fucose in the kidney during the critical period of ischaemia and reperfusion. The object would be to maximise the prevention of CL-11 binding to ligand at an appropriate stage of the surgical process. The precise timing and concentration L-fucose as well as route of administration remain to be established.

There are other therapeutic options to inhibit the lectin pathway, such as the targeting of lectin pathway proteases activated following CL-11 binding to ligand [49]. For example, monoclonal antibody against the protease MASP-2 has been successful in reducing lectin pathway-mediated inflammation in a variety of conditions [50, 51]. Because lectin molecules, besides CL-11, share MASPs, the use of MASP inhibitors given by the systemic route may affect a wider range of processes mediated by different C-type lectins, whereas the strategy to selectively block CL-11 in the transplant could have a more precise effect on the targeted inflammatory condition.

## Concluding remarks

**Fig. 2:**
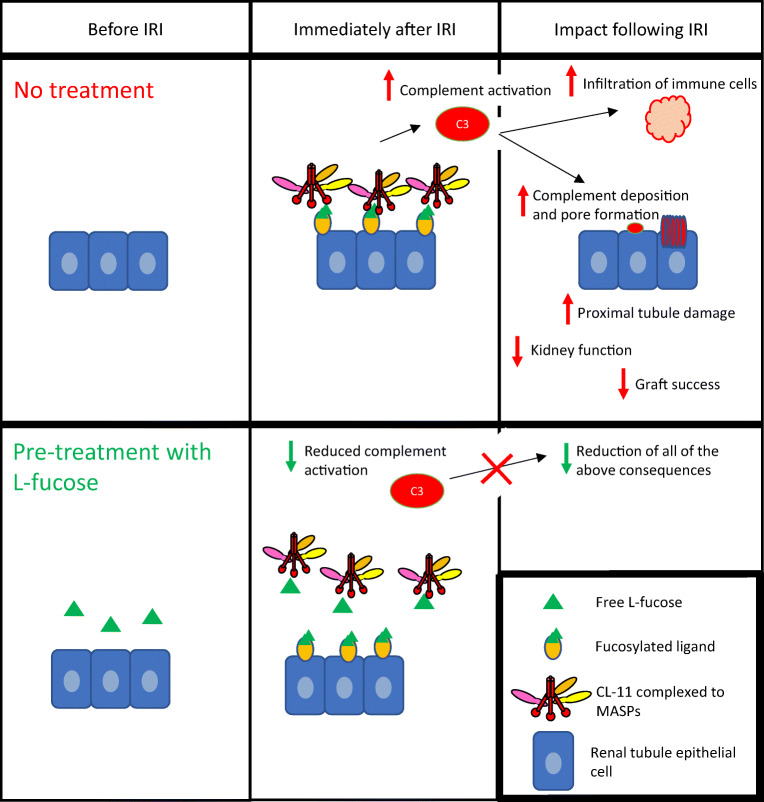
The effect of fucose treatment on complement signalling in renal ischaemia reperfusion injury (IRI). Without intervention, IRI causes the exposure of a fucosylated ligand on the surface of renal tubule cells. The lectin pathway pattern recognition molecule, CL-11, binds to this ligand and causes the activation of the complement system. This results in a number of detrimental effects for the kidney, in the first instance including complement deposition and the infiltration of immune cells. These consequences in turn result in kidney tubule damage and subsequent fibrosis, reduced kidney function and in the case of transplants a lower chance of graft success. Alternatively, the induction of a high level of L-fucose in the renal tubular space means that the carbohydrate recognition sites on CL-11 within this space are blocked. Therefore, following the IRI, the CL-11 can no longer bind to the exposed fucosylated ligand on the tubular surface and in turn does not activate complement. It is proposed that such treatment causes a downstream reduction in both the immediate and the longer-term functional consequences of complement activation in IRI

The finding of a new trigger mechanism in the induction of ischaemic injury that can be blocked by judicious use of L-fucose therapeutically is at an early stage of development. We recognise that before submitting the results to human investigation, a number of intermediate steps are required. This includes proof of concept in an animal model, such as a murine kidney transplant model, and possibly also with in vitro work on a human kidney tubule cell line. However, as a concept, it is worthy of discussion prior to this work being undertaken. The biochemical nature of the fucosylated ligand on damaged tissue and the precise timing of its interaction with CL-11 need to be more fully understood to allow potential clinical exploitation. However, in theory, a large enough increase in local L-fucose concentration within the kidney will block CL-11 binding and/or activation of complement, and in doing so will protect the donor kidney from the effects of IRI, and this has been shown in mice [48]. L-fucose monosaccharide is an excellent candidate for this because a number of studies have shown its safety at high levels in both mice and humans. For example, work in the cancer field has shown that murine intraperitoneal (IP) injection of L-fucose to a level of 5 g/kg can be administered without detrimental side effects [42]. Indeed, the work described here in IRI in mice supports this experience. In addition, the use of more complex saccharides such as low-molecular-weight-fucoidin (LMWF) obtained from brown seaweed by radical depolymerisation has been reported. When mice treated with 100 mg/kg/day of LMWF underwent IRI, they were protected from the associated tubular damage and reduced kidney function [52]. LMWF is rich in sulphated polysaccharides that are extracted from *L. japonica*. Other reports have characterised a wide variety of bioactivities, including anti-platelet aggregation and anti-inflammation [53]. Taking this finding into account alongside our work on CL-11, it is possible that soluble L-fucose or fucosylated compounds could produce clinically useful blockade of CL-11 made in the kidney and consequently reduce the complement mediated tubule damage associated with IRI. Furthermore, in treating patients with Leukocyte Adhesion Deficiency type II, serum levels of L-fucose up to 75× the normal level have been used to no ill effect [41, 54]. Together, these studies show that the short-term use of supraphysiological levels of L-fucose are unlikely to be an issue in terms of safety. A proposed scheme for the use of L-fucose to prevent complement-mediated injury in the process renal transplantation is summarised in Fig. 2. Our lab is currently undertaking studies to optimise the treatment protocol, for example to achieve extended exposure of the kidney to high-dose fucose through repeated injection of donor mice allowing for rapid clearance from the serum and kidney. The technique could also be beneficial to other donor organs, such as the liver, since raised hepatic levels of L-fucose also follow systemic administration [48]. Finally, the work on CL-11 and localised inhibitory concentrations of blocking sugar underpins the importance of tissue synthesis of complement and pattern recognition molecules. Treatment of the donor organ, as visualised here, has a number of potential advantages including high efficacy and low systemic side effects of localised treatment. L-fucose provides a conceptually simple and apparently safe approach worth exploring for the benefit of patients as well as validating the fundamental idea of CL-11 as a trigger mechanism for acute kidney injury.

## References

[1] Farrar CA, Tran D, Li K, Wu W, Peng Q, Schwaeble W, Zhou W, Sacks SH (2016) Collectin-11 detects stress-induced L-fucose pattern to trigger renal epithelial injury. J Clin Invest 126(5):1911–1925 10.1172/JCI83000 PMC4855924 27088797

[2] Wolfe RA, Ashby VB, Milford EL, Ojo AO, Ettenger RE, Agodoa LYC, Held PJ, Port FK (1999) Comparison of mortality in all patients on dialysis, patients on dialysis awaiting transplantation, and recipients of a first cadaveric transplant. N Engl J Med 341(23):1725–1730 10.1056/NEJM199912023412303 10580071

[3] Valderrabano F, Jofre R, Lopez-Gomez JM (2001). Quality of life in end-stage renal disease patients. Am J Kidney Dis.

[4] Rao PS, Ojo A (2009). The alphabet soup of kidney transplantation: SCD, DCD, ECD--fundamentals for the practicing nephrologist. Clin J Am Soc Nephrol.

[5] Lee LY, Pham TA, Melcher ML (2019). Living kidney donation: strategies to increase the donor pool. Surg Clin North Am.

[6] Bugeja A, Clark EG (2017). Living kidney donation. CMAJ.

[7] Orandi BJ, Luo X, Massie AB, Garonzik-Wang JM, Lonze BE, Ahmed R, Van Arendonk KJ, Stegall MD, Jordan SC, Oberholzer J, Dunn TB, Ratner LE, Kapur S, Pelletier RP, Roberts JP, Melcher ML, Singh P, Sudan DL, Posner MP, El-Amm JM, Shapiro R, Cooper M, Lipkowitz GS, Rees MA, Marsh CL, Sankari BR, Gerber DA, Nelson PW, Wellen J, Bozorgzadeh A, Gaber AO, Montgomery RA, Segev DL (2016) Survival benefit with kidney transplants from HLA-incompatible live donors. N Engl J Med 374(10):940–950 10.1056/NEJMoa1508380 PMC4841939 26962729

[8] Sacks SH, Zhou W (2012). The role of complement in the early immune response to transplantation. Nat Rev Immunol.

[9] Pratt JR, Basheer SA, Sacks SH (2002). Local synthesis of complement component C3 regulates acute renal transplant rejection. Nat Med.

[10] Marsh JE, Farmer CK, Jurcevic S, Wang Y, Carroll MC, Sacks SH (2001) The allogeneic T and B cell response is strongly dependent on complement components C3 and C4. Transplantation 72(7):1310–1318 10.1097/00007890-200110150-00022 11602861

[11] Fremeaux-Bacchi V, Legendre CM (2015). The emerging role of complement inhibitors in transplantation. Kidney Int.

[12] Stegall MD, Diwan T, Raghavaiah S, Cornell LD, Burns J, Dean PG, Cosio FG, Gandhi MJ, Kremers W, Gloor JM (2011) Terminal complement inhibition decreases antibody-mediated rejection in sensitized renal transplant recipients. Am J Transplant 11(11):2405–2413 10.1111/j.1600-6143.2011.03757.x 21942930

[13] Nauser CL, Howard MC, Fanelli G, Farrar CA, Sacks S (2018) Collectin-11 (CL-11) is a major sentinel at epithelial surfaces and key pattern recognition molecule in complement-mediated ischaemic injury. Front Immunol 9:2023 10.3389/fimmu.2018.02023 PMC6136055 30237800

[14] Farrar CA, Zhou W, Sacks SH (2016). Role of the lectin complement pathway in kidney transplantation. Immunobiology.

[15] Lee H, Han E, Choi AR, Ban TH, Chung BH, Yang CW, Choi YJ, Oh EJ (2018) Clinical impact of complement (C1q, C3d) binding de novo donor-specific HLA antibody in kidney transplant recipients. PLoS One 13(11):e0207434 10.1371/journal.pone.0207434 PMC6235372 30427941

[16] Zhou W, Farrar CA, Abe K, Pratt JR, Marsh JE, Wang Y, Stahl GL, Sacks SH (2000) Predominant role for C5b-9 in renal ischemia/reperfusion injury. J Clin Invest 105(10):1363–1371 10.1172/JCI8621 PMC315463 10811844

[17] Lin T, Zhou W, Farrar CA, Hargreaves RE, Sheerin NS, Sacks SH (2006) Deficiency of C4 from donor or recipient mouse fails to prevent renal allograft rejection. Am J Pathol 168(4):1241–1248 10.2353/ajpath.2006.050360 PMC1606553 16565498

[18] Howard M, Farrar CA, Sacks SH (2018). Structural and functional diversity of collectins and ficolins and their relationship to disease. Semin Immunopathol.

[19] Matsushita M, Fujita T (2002). The role of ficolins in innate immunity. Immunobiology.

[20] Garred P, Genster N, Pilely K, Bayarri-Olmos R, Rosbjerg A, Ma YJ, Skjoedt MO (2016) A journey through the lectin pathway of complement-MBL and beyond. Immunol Rev 274(1):74–97 10.1111/imr.12468 27782323

[21] Weis WI, Drickamer K, Hendrickson WA (1992). Structure of a C-type mannose-binding protein complexed with an oligosaccharide. Nature.

[22] Drickamer K (1992). Engineering galactose-binding activity into a C-type mannose-binding protein. Nature.

[23] Hansen S, Selman L, Palaniyar N, Ziegler K, Brandt J, Kliem A, Jonasson M, Skjoedt MO, Nielsen O, Hartshorn K, Jørgensen TJ, Skjødt K, Holmskov U (2010) Collectin 11 (CL-11, CL-K1) is a MASP-1/3-associated plasma collectin with microbial-binding activity. J Immunol 185(10):6096–6104 10.4049/jimmunol.1002185 20956340

[24] Venkatraman Girija U, Furze CM, Gingras AR, Yoshizaki T, Ohtani K, Marshall JE, Wallis AK, Schwaeble WJ, El-Mezgueldi M, Mitchell DA, Moody P, Wakamiya N, Wallis R (2015) Molecular basis of sugar recognition by collectin-K1 and the effects of mutations associated with 3MC syndrome. BMC Biol 13:27 10.1186/s12915-015-0136-2 PMC4431178 25912189

[25] Hansen SWK, Aagaard JB, Bjerrum KB, Hejbøl EK, Nielsen O, Schrøder HD, Skjoedt K, Sørensen AL, Graversen JH, Henriksen ML (2018) CL-L1 and CL-K1 exhibit widespread tissue distribution with high and co-localized expression in secretory epithelia and mucosa. Front Immunol 9:1757 10.3389/fimmu.2018.01757 PMC6079254 30108587

[26] Schwaeble WJ, Lynch NJ, Clark JE, Marber M, Samani NJ, Ali YM, Dudler T, Parent B, Lhotta K, Wallis R, Farrar CA, Sacks S, Lee H, Zhang M, Iwaki D, Takahashi M, Fujita T, Tedford CE, Stover CM (2011) Targeting of mannan-binding lectin-associated serine protease-2 confers protection from myocardial and gastrointestinal ischemia/reperfusion injury. Proc Natl Acad Sci U S A 108(18):7523–7528 10.1073/pnas.1101748108 PMC3088599 21502512

[27] Morgan BP, Boyd C, Bubeck D (2017). Molecular cell biology of complement membrane attack. Semin Cell Dev Biol.

[28] Mathern DR, Heeger PS (2015). Molecules great and small: the complement system. Clin J Am Soc Nephrol.

[29] Hearse DJ, Bolli R (1992). Reperfusion induced injury: manifestations, mechanisms, and clinical relevance*. Cardiovasc Res.

[30] Asgari E, Farrar CA, Lynch N, Ali YM, Roscher S, Stover C, Zhou W, Schwaeble WJ, Sacks SH (2014) Mannan-binding lectin-associated serine protease 2 is critical for the development of renal ischemia reperfusion injury and mediates tissue injury in the absence of complement C4. FASEB J 28(9):3996–4003 10.1096/fj.13-246306 PMC5184842 24868011

[31] Thurman JM, Royer PA, Ljubanovic D, Dursun B, Lenderink AM, Edelstein CL, Holers VM (2006) Treatment with an inhibitory monoclonal antibody to mouse factor B protects mice from induction of apoptosis and renal ischemia/reperfusion injury. J Am Soc Nephrol 17(3):707–715 10.1681/ASN.2005070698 16467447

[32] Kosieradzki, M. and W. Rowiński. *Ischemia/reperfusion injury in kidney transplantation: mechanisms and prevention*. in *Transplantation proceedings*. 2008. Elsevier 10.1016/j.transproceed.2008.10.004 19100373

[33] Thurman JM (2007). Triggers of inflammation after renal ischemia/reperfusion. Clin Immunol.

[34] Farrar CA, Zhou W, Lin T, Sacks SH (2006) Local extravascular pool of C3 is a determinant of postischemic acute renal failure. FASEB J 20(2):217–226 10.1096/fj.05-4747com 16449793

[35] Yaseen S, Demopulos G, Dudler T, Yabuki M, Wood CL, Cummings WJ, Tjoelker LW, Fujita T, Sacks S, Garred P, Andrew P, Sim RB, Lachmann PJ, Wallis R, Lynch N, Schwaeble WJ (2017) Lectin pathway effector enzyme mannan-binding lectin-associated serine protease-2 can activate native complement C3 in absence of C4 and/or C2. FASEB J 31(5):2210–2219 10.1096/fj.201601306R 28188176

[36] Hart ML, Ceonzo KA, Shaffer LA, Takahashi K, Rother RP, Reenstra WR, Buras JA, Stahl GL (2005) Gastrointestinal ischemia-reperfusion injury is lectin complement pathway dependent without involving C1q. J Immunol 174(10):6373–6380 10.4049/jimmunol.174.10.6373 15879138

[37] Walsh MC, Bourcier T, Takahashi K, Shi L, Busche MN, Rother RP, Solomon SD, Ezekowitz RA, Stahl GL (2005) Mannose-binding lectin is a regulator of inflammation that accompanies myocardial ischemia and reperfusion injury. J Immunol 175(1):541–546 10.4049/jimmunol.175.1.541 15972690

[38] Keshi H, Sakamoto T, Kawai T, Ohtani K, Katoh T, Jang SJ, Motomura W, Yoshizaki T, Fukuda M, Koyama S, Fukuzawa J, Fukuoh A, Yoshida I, Suzuki Y, Wakamiya N (2006) Identification and characterization of a novel human collectin CL-K1. Microbiol Immunol 50(12):1001–1013 10.1111/j.1348-0421.2006.tb03868.x 17179669

[39] Becker DJ, Lowe JB (2003). Fucose: biosynthesis and biological function in mammals. Glycobiology.

[40] Schneider M, Al-Shareffi E, Haltiwanger RS (2017). Biological functions of fucose in mammals. Glycobiology.

[41] Wild MK1, Lühn K, Marquardt T, Vestweber D (2002) Leukocyte adhesion deficiency II: therapy and genetic defect. Cells Tissues Organs 172(3):161–173 10.1159/000066968 12476046

[42] Tomsik P, Soukup T, Cermakova E, Micuda S, Niang M, Sucha L, Rezacova M (2011) L-rhamnose and L-fucose suppress cancer growth in mice. Central European Journal of Biology 6(1):1–9

[43] Kaneko M, Kudo T, Iwasaki H, Ikehara Y, Nishihara S, Nakagawa S, Sasaki K, Shiina T, Inoko H, Saitou N, Narimatsu H (1999) Alpha1,3-fucosyltransferase IX (Fuc-TIX) is very highly conserved between human and mouse; molecular cloning, characterization and tissue distribution of human Fuc-TIX. FEBS Lett 452(3):237–242 10.1016/s0014-5793(99)00640-7 10386598

[44] Werz DB, Ranzinger R, Herget S, Adibekian A, von der Lieth CW, Seeberger PH (2007) Exploring the structural diversity of mammalian carbohydrates ("glycospace") by statistical databank analysis. ACS Chem Biol 2(10):685–691 10.1021/cb700178s 18041818

[45] Stanley, P., N. Taniguchi, and M. Aebi, *N-Glycans*, in *Essentials of Glycobiology*, rd, et al., Editors. 2015: Cold Spring Harbor (NY). p. 99–111

[46] Luo Y, Nita-Lazar A, Haltiwanger RS (2006). Two distinct pathways for O-fucosylation of epidermal growth factor-like or thrombospondin type 1 repeats. J Biol Chem.

[47] Luo Y, Koles K, Vorndam W, Haltiwanger RS, Panin VM (2006) Protein O-fucosyltransferase 2 adds O-fucose to thrombospondin type 1 repeats. J Biol Chem 281(14):9393–9399 10.1074/jbc.M511975200 16464857

[48] Howard MC, Nauser CL, Farrar CA, Wallis R, Sacks SH (2020) L-Fucose prevention of renal ischaemia/reperfusion injury in mice. FASEB J 34(1):822–834 10.1096/fj.201901582R PMC6972607 31914693

[49] Kocsis A, Kékesi KA, Szász R, Végh BM, Balczer J, Dobó J, Závodszky P, Gál P, Pál G (2010) Selective inhibition of the lectin pathway of complement with phage display selected peptides against mannose-binding lectin-associated serine protease (MASP)-1 and -2: significant contribution of MASP-1 to lectin pathway activation. J Immunol 185(7):4169–4178 10.4049/jimmunol.1001819 20817870

[50] Kasanmoentalib SE, Valls Seron M, Ferwerda B, Tanck MW, Zwinderman AH, Baas F, van der Ende A, Brouwer MC, van de Beek D (2017) Mannose-binding lectin-associated serine protease 2 (MASP-2) contributes to poor disease outcome in humans and mice with pneumococcal meningitis. J Neuroinflammation 14(1):2 10.1186/s12974-016-0770-9 PMC5234106 28086930

[51] Clark JE, Dudler T, Marber MS, Schwaeble W (2018) Cardioprotection by an anti-MASP-2 antibody in a murine model of myocardial infarction. Open Heart 5(1):e000652 10.1136/openhrt-2017-000652 PMC5761301 29344374

[52] Chen J, Wang W, Zhang Q, Li F, Lei T, Luo D, Zhou H, Yang B (2013) Low molecular weight fucoidan against renal ischemia-reperfusion injury via inhibition of the MAPK signaling pathway. PLoS One 8(2):e56224 10.1371/journal.pone.0056224 PMC3572020 23418539

[53] Wang Z, Liu T, Chen X, You H, Zhang Q, Xue J, Zheng Y, Luo D (2018) Low molecular weight fucoidan ameliorates hindlimb ischemic injury in type 2 diabetic rats. J Ethnopharmacol 210:434–442 10.1016/j.jep.2017.09.014 28917976

[54] Marquardt T, Lühn K, Srikrishna G, Freeze HH, Harms E, Vestweber D (1999) Correction of leukocyte adhesion deficiency type II with oral fucose. Blood 94(12):3976–3985 10590041

